# Can Social Connections Improve Dementia Knowledge Among Homebound Community-Dwelling Older African Americans?

**DOI:** 10.1093/geroni/igaa057.930

**Published:** 2020-12-16

**Authors:** Maritza Dowling, Hiroko Dodge, Antonio Puente, Beverly Lunsford

**Affiliations:** 1 George Washington University - School of Nursing, Washington, District of Columbia, United States; 2 Pregon Health & Science University, Portland, Oregon, United States; 3 School of Medicine & Health Sciences, Washington, District of Columbia, United States; 4 George Washington University-School of Nursing, Washington, District of Columbia, United States

## Abstract

Despite the clear and compelling association between social connections and well-being, the underlying mechanisms that help stave off adverse health impacts are not well understood, particularly among older adults in disadvantaged groups. Social relationships in older age may be instrumental for exchanging and gaining knowledge that further influence health and help increase awareness about misconceptions and lifestyle behaviors known to delay or reduce cognitive decline. This study used cross-sectional survey data from 147(aged 58-90 years; 75% female) low-income African American homebound community dwellers to investigate heterogeneity in dementia literacy profiles and its association with social connectedness. Eleven items (false=0, true=1, don’t know=2) from a validated instrument were used to measure dementia literacy (DL). The Lubben’s social network scale was used for a social connectedness construct. We employed a 2-stage latent class modeling approach to examine heterogeneity in DL and estimate the regressions among the derived classes and the predictors (social connectedness, education level and age). A 3-class model produced a reasonable fit and classification (entropy=0.852) of “dementia literacy patterns” labeled as (high-literacy:37%), moderate-literacy:45.2%), low-literacy:17.8%)). Social connectedness was highly predictive of class membership. A high level of social relationships increased the probability of being in the “high-dementia-literacy” class compared to the “low-dementia-literacy” class (OR=2.189, p=0.016). For a unit increase in social connectedness, the odds of being in the “high-dementia-literacy” class compared to the “low-dementia-literacy” class increased by a factor of 2.2. Tailored and focused interventions to reduce social disconnectedness may also help increase dementia awareness and reduce barriers to early diagnosis.

